# Associations of systemic inflammation response index and systemic immune-inflammation index with proxy-assessed sarcopenia in community-dwelling older adults

**DOI:** 10.3389/fmed.2026.1764196

**Published:** 2026-05-05

**Authors:** Min Tang, Zhihan Hu, Rong Wu, Qin Liu, Tengfei Wu, Xinyu Yang, Liping Hu, Yilei Cong, Hua Yang

**Affiliations:** 1 Department of Endocrinology, Longhua Hospital, Shanghai University of Traditional Chinese Medicine, Shanghai, China; 2 Department of Nursing, Longhua Hospital, Shanghai University of Traditional Chinese Medicine, Shanghai, China; 3 Department of Internal Medicine, Datuan Community Health Center, Shanghai, China

**Keywords:** inflammation, older adults, sarcopenia, systemic immune-inflammation index, systemic inflammation response index

## Abstract

**Objective:**

Sarcopenia is a progressive and complex disorder associated with higher morbidity and mortality, which significantly increases the financial burden and medical costs. This study aims to investigate the relationship of the systemic inflammation response index (SIRI) and the systemic immune-inflammation index (SII) with proxy-assessed sarcopenia.

**Methods:**

This study was a cross-sectional and observational study. The sociodemographic information, lifestyle habits, anthropometric measurements, disease history and medication history, and medical examination data of these individuals were collected. Multivariate logistic regression analyses were performed to estimate the odds ratio (ORs) and 95% confidence intervals (CIs) for proxy-assessed sarcopenia when adjusted for different confounders. Restricted cubic spline (RCS) regression model was used to model the association of SIRI or SII with the presence of proxy-assessed sarcopenia. Furthermore, the associations between SIRI or SII and proxy-assessed sarcopenia in different subgroups by grouping sex, age, metabolic dysfunction-associated steatotic liver disease (MASLD), current smoking, alcohol consumption, type 2 diabetes mellitus, hypertension, dyslipidemia were identified by multivariate logistic regression models.

**Results:**

A total of 1706 community-dwelling older adults participated in this study, including 949 males and 757 females, with a median age of 72 years for the total population. In the multivariate logistic regression models, after adjusting for confounding factors, a statistically significant association with SIRI or SII and proxy-assessed sarcopenia was found. The RCS model showed that a positive association between SIRI and proxy-assessed sarcopenia, as well as SII and proxy-assessed sarcopenia, and the non-linear relationship between SIRI and proxy-assessed sarcopenia was statistically significant. Similar results were found in the association between SII and proxy-assessed sarcopenia.

**Conclusion:**

In conclusion, our study demonstrated a significant association of SIRI and SII with proxy-assessed sarcopenia in community-dwelling older adults, underscoring the relevance of systemic inflammation in proxy-assessed sarcopenia and supporting the potential value of these indices in future pathophysiological and longitudinal studies.

## Introduction

Sarcopenia, characterized by the progressive loss of skeletal muscle mass and function, is regarded to be strongly associated with multiple adverse clinical outcomes (1), representing a significant health concern, particularly among the aging population. Sarcopenia increases the risk of disability, falls and fall-related injuries, impaired quality of life, hospitalization, loss of independence, and mortality, making it one of the major health problems in older adults (2–4), and a significant public health challenge in an increasingly aging society. Although the prevalence of sarcopenia may vary by definition and study population (5), the prevalence of sarcopenia among community-dwelling older persons is estimated to range from 9.9% to 40.4% (6). Despite the growing recognition of sarcopenia’s impact, current therapeutic options remain limited. Currently, no drugs have been approved by the Food and Drug Administration for the treatment of sarcopenia (7). The lack of effective treatments underscores the importance of early detection and prevention strategies. Identifying individuals at risk and implementing lifestyle modifications, such as resistance training and balanced nutrition, can potentially mitigate the progression of sarcopenia and improve outcomes.

Although the pathophysiology of sarcopenia is multifactorial, a growing body of research emphasizes inflammation as a key regulator of the homeostatic mechanisms that control skeletal muscle homeostasis and ultimately lead to muscle atrophy (8–10). Inflammatory markers, such as C-reactive protein (CRP), interleukin-6 (IL-6), and tumor necrosis factor-alpha (TNFα) are often elevated in individuals with sarcopenia, suggesting that systemic inflammation may drive muscle wasting and impair muscle function (8). In recent years, novel systemic inflammatory indices derived from routine blood counts–such as the systemic inflammation response index (SIRI) and the systemic immune-inflammation index (SII)–have emerged as promising and easily accessible biomarkers. SIRI, which integrates neutrophil, monocyte, and lymphocyte counts to provide a comprehensive assessment of the body’s inflammatory status, was initially used to predict survival in patients with pancreatic adenocarcinoma receiving chemotherapy (11). Previous studies have highlighted the potential utility of SIRI in predicting various clinical outcomes (12–16). SII, calculated from platelet, neutrophil, and lymphocyte counts, was created in 2014 to reflect local immune response and systemic inflammation and predict prognosis of patients after curative resection for hepatocellular carcinoma (17). SII has demonstrated prognostic value in various chronic diseases (18–20). SII and SIRI are showing potential associations with acute coronary syndrome, coronary artery disease severity, hypertension and cardiometabolic multimorbidity, though their specific roles vary across different patient populations (21–24). A previous study has compared SIRI and SII with traditional inflammatory markers in predicting a favorable response to omalizumab in patients with chronic spontaneous urticaria, supporting their utility as surrogate markers of systemic inflammation (25). Therefore, these composite indices may offer a practical, cost-effective alternative to direct cytokine measurements in routine clinical practice. However, their specific association with sarcopenia in community-dwelling older adults remains underexplored.

In this study, we aim to determine whether SIRI and SII are independently associated with proxy-assessed sarcopenia and explore their potential value as biomarkers for early detection. By elucidating the association of SIRI and SII with proxy-assessed sarcopenia, we hope to provide insights that may inform future research into preventive and therapeutic strategies for this debilitating condition.

## Materials and methods

### Study design

This study was an observational and cross-sectional study. A total of 1706 community-dwelling older adults were enrolled in October 2024. This study was conducted in accordance with the Declaration of Helsinki. All participants signed the informed consent before participating. The study was approved by the Ethics Committee of Longhua Hospital, Shanghai University of TCM (2024LCSY075).

#### Study population

The study population was drawn from the Datuan community in Shanghai, China. Data on lifestyle habits, disease history, medication history, health check-ups, and physical examinations were collected. A total of 1706 community-dwelling older adults meeting the inclusion criteria were included in the study. The inclusion criteria were as follows: willingness to participate in the study, and age ≥ 65 years. The exclusion criteria were as follows: older adults with neuromuscular disorders affecting limb function and balance; older adults with impaired balance; older adults with arthritis of the lower extremities; older adults with chronic cardiopulmonary insufficiency; older adults with consumptive diseases such as malignant tumors, chronic gastrointestinal diseases, or infectious diseases; older adults with acute illnesses (e.g., acute infections, acute organ failure); and older adults unable to cooperate in completing questionnaires or instructions.

#### Study data collection

A standardized questionnaire was used to assist staff in gathering data on lifestyle habits, disease history, and medication history, while medical examination data were also collected. Health check-up data were collected, including sex, age, height, weight, body mass index (BMI), waist circumference, hemoglobin, erythrocyte count, leukocyte count, neutrophil count, monocyte count, basophil count, eosinophil count, lymphocyte count, platelet count, urea nitrogen, creatinine, fasting plasma glucose (FPG), total bilirubin, triglyceride, total cholesterol, alanine transaminase, aspartate aminotransferase, high-density lipoprotein cholesterol (HDL-C), and low-density lipoprotein cholesterol (LDL-C). Physical examinations were performed according to a standardized protocol, including measurements of calf circumference, grip strength, five-times sit-to-stand, and 6-meter gait speed. Calf circumference was measured by wrapping a tape measure around the thickest part of the calf. Grip strength was measured using an electronic grip strength instrument (Xiangshan CAMRY EH101). The five-times sit-to-stand test was performed at the fastest possible speed. Gait speed was measured by asking participants to walk a 6-meter straight line at their usual pace; the time was recorded twice, and the average was used to calculate gait speed (m/s).

### Assessment of sarcopenia

Sarcopenia was diagnosed according to the 2019 criteria of the Asian Working Group for Sarcopenia (AWGS), which require the presence of low muscle strength, low muscle mass, and/or low physical performance (26). Low muscle strength was assessed by measuring handgrip strength (<18 kg for female and <28 kg for male). Muscle mass in this study was estimated using a validated population-specific formula: ASM (kg) = 0.193 × weight (kg) + 0.107 × height (cm) − 4.157 × gender − 0.037 × age (years) − 2.631, where gender was coded as 1 for males and 2 for females (27). Low muscle mass was defined as a height-adjusted appendicular skeletal muscle mass (ASM/Ht^2^) below sex-specific thresholds (<5.27 kg/m^2^ for women and <7.03 kg/m^2^ for men), corresponding to the lowest 20% of the distribution in the study population. Given that muscle mass was estimated using a population-specific prediction equation rather than direct imaging, the diagnosis of sarcopenia in this study is proxy-assessed sarcopenia. Physical performance was evaluated using the five-times sit-to-stand test and 6-meter gait speed. Low physical performance was defined as a five-times sit-to-stand time ≥ 12 s or a gait speed < 1.0 m/s. According to the AWGS 2019 diagnostic criteria, individuals diagnosed with both low grip strength and low muscle mass were diagnosed with sarcopenia. If low physical performance was also identified, the diagnosis can be classified as severe sarcopenia (28).

#### Definition of variables

The definition of hypertension was DBP ≥ 90 mmHg and/or SBP ≥ 140 mmHg following repeated examination (29) or prior diagnosis of hypertension by a physician. Type 2 diabetes mellitus was defined as a prior diagnosis of type 2 diabetes mellitus by a physician. Dyslipidemia was defined as a prior diagnosis of dyslipidemia by a physician. SIRI was calculated using the following formula: SIRI = (neutrophil count × monocyte count)/lymphocyte count. SII was calculated as the following formula: (platelet count × neutrophil count)/lymphocyte count.

### Statistical analyses

Data were presented as numbers or medians with interquartile ranges. For continuous variables with a skewed distribution, the Mann–Whitney U test was used, while the Chi-square test was applied to assess differences between groups for categorical variables. To assess the independent association of SIRI and SII with proxy-assessed sarcopenia, multivariate logistic regression models were employed. A two-sided *P*-value of less than 0.05 was considered to indicate a statistically significant difference between the two groups.

Moreover, multivariate logistic regression analyses were performed to estimate the odds ratios (ORs) and corresponding 95% confidence intervals (CIs) for proxy-assessed sarcopenia when adjusted for different confounders. Restricted cubic spline (RCS) models were fitted with three knots at the 5th, 50th, and 95th percentiles of SIRI and SII to model their association with proxy-assessed sarcopenia. Subgroup analyses were performed using multivariate logistic regression models to evaluate the associations between SIRI or SII and proxy-assessed sarcopenia across subgroups stratified by sex, age, MASLD, current smoking, alcohol consumption, type 2 diabetes mellitus, hypertension, and dyslipidemia. The ORs and 95% CIs for proxy-assessed sarcopenia were calculated accordingly. Furthermore, the interactions between these subgroup variables and SIRI or SII were assessed. Our study’s statistical analyses were performed using IBM SPSS (version 27.0) and R statistical software (version 4.5.0).

## Results

### Baseline characteristics of study individuals

A total of 1706 community-dwelling individuals participated in this study, including 949 males and 757 females, with a median age of 72 years for the total population. Of the participants, 279 were diagnosed with proxy-assessed sarcopenia. Comparative analysis revealed a statistically significant difference in SII between the proxy-assessed sarcopenia and non-proxy-assessed sarcopenia groups, with higher values observed in the former. For SIRI, however, the difference between the groups was not statistically significant. Other variables that differed significantly between the groups included age, sex, weight, height, BMI, waist circumference, hemoglobin, erythrocyte count, leukocyte count, lymphocyte count, monocyte count, eosinophil count, platelet count, creatinine, FPG, triglyceride, total bilirubin, alanine transaminase, HDL-C, hypertension, dyslipidemia, MASLD, and type 2 diabetes mellitus. The baseline characteristics of the study population with and without proxy-assessed sarcopenia were summarized in Table 1.

**TABLE 1:** Baseline characteristics of the study subjects.

Variables	No proxy-assessed sarcopenia (*n* = 1427; 83.65%)	Proxy-assessed sarcopenia (*n* = 279; 16.35%)	*P*-value
Age (years)	72 (69–76)	75 (71–80)	<0.001
Height (cm)	161.0 (155.5–167.0)	157.0 (151.0–163.0)	<0.001
Weight (kg)	65.0 (58.5–71.0)	48.0 (45.5–52.0)	<0.001
BMI (kg/m^2^)	24.7 (23.1–26.7)	19.9 (18.9–20.7)	<0.001
Waist circumference (cm)	85.0 (80.0–90.0)	74.0 (70.0–78.0)	<0.001
Erythrocyte count (10^12/L)	4.66 (4.35–4.96)	4.40 (4.10–4.72)	<0.001
Hemoglobin (g/L)	142 (133–151)	133 (124–143)	<0.001
Leukocyte count (10^9/L)	6.29 (5.34–7.35)	5.99 (4.89–7.22)	0.003
Neutrophil count (10^9/L)	3.52 (2.88–4.32)	3.46 (2.75–4.37)	0.595
Monocyte count (10^9/L)	0.42 (0.34–0.51)	0.40 (0.32–0.48)	0.007
Basophil count (10^9/L)	0.03 (0.02–0.04)	0.03 (0.02–0.04)	0.450
Eosinophil count (10^9/L)	0.12 (0.07–0.18)	0.10 (0.06–0.17)	0.004
Lymphocyte count (10^9/L)	2.06 (1.67–2.49)	1.81 (1.42–2.30)	<0.001
Platelet count (10^9/L)	205 (170–244)	214 (179–249)	0.020
ALT (IU/L)	19 (15–26)	16 (12–19)	<0.001
AST (IU/L)	23 (20–27)	23 (20–26)	0.092
FPG (mmol/L)	5.83 (5.34–6.66)	5.46 (5.16–5.94)	<0.001
Urea nitrogen (mmol/L)	6.4 (5.4–7.6)	6.6 (5.6–7.7)	0.315
Creatinine (μmol/L)	72 (61–86)	69 (58–81)	0.018
Total bilirubin (μmol/L)	14.1 (11.0–18.0)	13.2 (10.7–16.2)	0.008
Triglyceride (mmol/L)	1.29 (0.93–1.81)	0.96 (0.75–1.28)	<0.001
Total cholesterol (mmol/L)	5.0 (4.2–5.7)	5.1 (4.4–5.8)	0.059
SIRI	0.693 (0.505–1.017)	0.751 (0.509–1.101)	0.117
SII	347.3 (253.5–474.7)	398.2 (278.6–575.7)	<0.001
HDL-C (mmol/L)	1.25 (1.05–1.49)	1.51 (1.29–1.73)	<0.001
LDL-C (mmol/L)	2.75 (2.02–3.36)	2.70 (2.10–3.25)	0.480
Grip strength (kg)	14.4 (9.7–20.2)	11.5 (7.4–15.8)	<0.001
Calf circumference (cm)	33.7 (31.9–35.5)	30.3 (29.0–31.9)	<0.001
Five times sit to stand (s)	11.46 (9.2–14.34)	12.08 (10.01–14.80)	0.017
Gait speed (m/s)	0.84 (0.67–1.03)	0.76 (0.63–0.95)	<0.001
ASM (kg)	17.57 (14.60–21.61)	12.46 (11.22–17.72)	<0.001
ASM/Ht^2^ (kg/m^2^)	6.99 (5.95–7.73)	5.17 (4.91–6.63)	<0.001
Sex (male/female)	652/775	105/174	0.016
Current smoking (no/yes)	1186/241	236/43	0.605
Alcohol consumption (no/yes)	1229/198	249/30	0.192
Type 2 diabetes mellitus (no/yes)	1152/275	262/17	<0.001
Hypertension (no/yes)	554/873	164/115	<0.001
Dyslipidemia (no/yes)	1232/195	267/12	<0.001
MASLD (no/yes)	691/736	259/20	<0.001

Data were N or median (interquartile range). Continuous variables used Mann-Whitney U test and categorical variables used chi-squared test for comparing the baseline characteristics of older adults with proxy-assessed sarcopenia or without proxy-assessed sarcopenia. BMI, body mass index; FPG, fasting plasma glucose; ALT, alanine transaminase; AST, aspartate aminotransferase; HDL-C, high-density lipoprotein cholesterol; LDL-C, low-density lipoprotein cholesterol; MASLD, metabolic dysfunction-associated steatotic liver disease; ASM, appendicular skeletal muscle mass; SIRI, systemic inflammation response index; SII, systemic immune-inflammation index.

#### Association between SIRI, SII and proxy-assessed sarcopenia

In the multivariate logistic regression models, factors independently associated with proxy-assessed sarcopenia were identified after adjusting for potential confounders. After categorizing participants into quartiles based on SIRI or SII levels (Q1: lowest, Q4: highest), respectively, a clear dose-response relationship was observed. In the multivariate logistic regression model adjusted for waist circumference, current smoking, alcohol consumption, hemoglobin, alanine transaminase, aspartate aminotransferase, fasting plasma glucose, total cholesterol, total bilirubin, triglyceride, creatinine, erythrocyte count, HDL-C, LDL-C, MASLD, type 2 diabetes mellitus, hypertension, dyslipidemia, sex and age, the odds of proxy-assessed sarcopenia increased significantly across ascending SIRI quartiles or SII quartiles, respectively. Compared to the lowest quartile (Q1) of SIRI as the reference, the adjusted ORs and 95% CIs for Q2, Q3, and Q4 were 1.587 (0.939–2.683), 2.033 (1.208–3.422) and 2.313 (1.354–3.950), respectively, and the P for trend across quartiles was 0.012 (Table 2). Compared to the Q1 of SII as the reference, the adjusted ORs and 95% CIs for Q2, Q3, and Q4 were 1.512 (0.869–2.631), 2.033 (1.179–3.505), 3.430 (2.022–5.819), respectively, and the P for trend across quartiles was <0.001 (Table 3).

**TABLE 2:** Relations of systemic inflammation response index with proxy-assessed sarcopenia.

SIRI					
Model	Q1 (*N* = 427)	Q2 (*N* = 426)	Q3 (*N* = 427)	Q4 (*N* = 426)	*P*-value for trend
Model 1 - OR (95% CI)	Ref.	1.236 (0.776–1.970)	1.744 (1.112–2.737)	2.027 (1.287–3.191)	0.009
Model 2 - OR (95% CI)	Ref.	1.230 (0.772–1.962)	1.709 (1.087–2.687)	1.979 (1.253–3.127)	0.015
Model 3 - OR (95% CI)	Ref.	1.365 (0.841–2.215)	2.051 (1.277–3.294)	2.502 (1.536–4.077)	0.001
Model 4 - OR (95% CI)	Ref.	1.587 (0.939–2.683)	2.033 (1.208–3.422)	2.313 (1.354–3.950)	0.012

Model 1 was adjusted for waist circumference. Model 2 was further adjusted for current smoking and alcohol consumption. Model 3 was further adjusted for hemoglobin, alanine transaminase, aspartate aminotransferase, fasting plasma glucose, total cholesterol, total bilirubin, triglyceride, creatinine, erythrocyte count, high-density lipoprotein cholesterol, and low-density lipoprotein cholesterol. Model 4 was further adjusted for MASLD, type 2 diabetes mellitus, hypertension, dyslipidemia, sex and age. The quartile ranges of Q1, Q2, Q3, and Q4 of SIRI were <0.502, 0.502–0.704, 0.704–1.025, >1.025, respectively. Q1 is the reference group. Multivariate logistic regression analyses were performed to estimate the ORs and corresponding 95% CIs for proxy-assessed sarcopenia. Q, quartile; OR, odds ratio; CI, confidence interval; N, number; MASLD, metabolic dysfunction-associated steatotic liver disease; SIRI, systemic inflammation response index.

**TABLE 3:** Relations of systemic immune-inflammation index with proxy-assessed sarcopenia.

SII					
Model	Q1 (*N* = 427)	Q2 (*N* = 426)	Q3 (*N* = 427)	Q4 (*N* = 426)	*P*-value for trend
Model 1 - OR (95% CI)	Ref.	1.218 (0.745–1.990)	1.486 (0.923–2.393)	2.676 (1.684–4.252)	<0.001
Model 2 - OR (95% CI)	Ref.	1.187 (0.725–1.943)	1.448 (0.897–2.335)	2.627 (1.652–4.178)	<0.001
Model 3 - OR (95% CI)	Ref.	1.346 (0.805–2.248)	1.851 (1.117–3.064)	3.234 (1.986–5.265)	<0.001
Model 4 - OR (95% CI)	Ref.	1.512 (0.869–2.631)	2.033 (1.179–3.505)	3.430 (2.022–5.819)	<0.001

Model 1 was adjusted for waist circumference. Model 2 was further adjusted for current smoking and alcohol consumption. Model 3 was further adjusted for hemoglobin, alanine transaminase, aspartate aminotransferase, fasting plasma glucose, total cholesterol, total bilirubin, triglyceride, creatinine, erythrocyte count, high-density lipoprotein cholesterol, and low-density lipoprotein cholesterol. Model 4 was further adjusted for MASLD, type 2 diabetes mellitus, hypertension, dy sex slipidemia, and age. The quartile ranges of Q1, Q2, Q3, and Q4 of SII were <258.824, 258.824–357.127, 357.127–488.098, >488.098, respectively. Q1 is the reference group. Multivariate logistic regression analyses were performed to estimate the ORs and corresponding 95% CIs for proxy-assessed sarcopenia. Q, quartile; OR, odds ratio; CI, confidence interval; N, number; MASLD, metabolic dysfunction-associated steatotic liver disease; SII, systemic immune-inflammation index.

**FIGURE 1:**
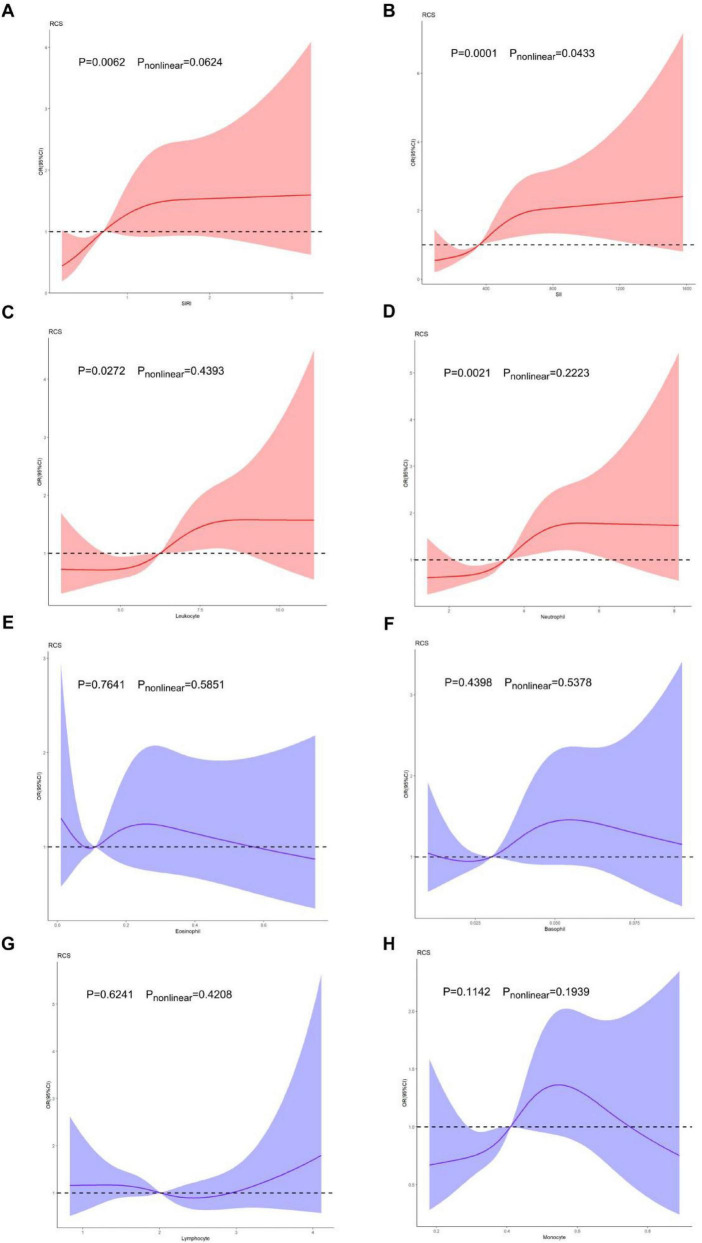
Restricted cubic spline models. Restricted cubic spline models were adopted to demonstrate dose–response associations between SIRI **(A)**, SII **(B)**, leukocyte **(C)**, neutrophil **(D)**, eosinophil **(E)**, basophil **(F)**, lymphocyte **(G)**, monocyte **(H)** and the prevalence of proxy-assessed sarcopenia; RCS models were adjusted for sex, age, waist circumference, current smoking, alcohol consumption, hemoglobin, alanine transaminase, aspartate aminotransferase, fasting plasma glucose, total cholesterol, total bilirubin, triglyceride, creatinine, erythrocyte count, high-density lipoprotein cholesterol, low-density lipoprotein cholesterol, MASLD, type 2 diabetes mellitus, hypertension and dyslipidemia. OR, odds ratio; CI, confidence interval; RCS, restricted cubic splines; SIRI, systemic inflammation response index; SII, systemic immune-inflammation index.

The dose-response relationship between various hematological inflammatory parameters (including SIRI, SII, leukocyte count, neutrophil count, monocyte count, basophil count, eosinophil count, lymphocyte count) and the odds of proxy-assessed sarcopenia was further elucidated using RCS analysis, after adjusting for sex, age, waist circumference, current smoking, alcohol consumption, hemoglobin, alanine transaminase, aspartate aminotransferase, fasting plasma glucose, total cholesterol, total bilirubin, triglyceride, creatinine, erythrocyte count, HDL-C, LDL-C, MASLD, type 2 diabetes mellitus, hypertension and dyslipidemia. Among the parameters tested, only SIRI, SII, leukocyte count and neutrophil count demonstrated statistically significant overall associations with the odds of proxy-assessed sarcopenia (all *P* for overall association < 0.05). Crucially, when examining the shape of these associations, only SIRI and SII exhibited significant non-linearity (*P* for non-linearity < 0.05). The RCS curves for SIRI and SII indicated a non-linear, positive dose-response relationship, where the odds of proxy-assessed sarcopenia increased progressively but at a non-constant rate across the range of values. No significant associations were observed for the remaining cell counts (all P for overall association > 0.05). The RCS models are presented in Figure 1.

## Subgroup analyses

**FIGURE 2:**
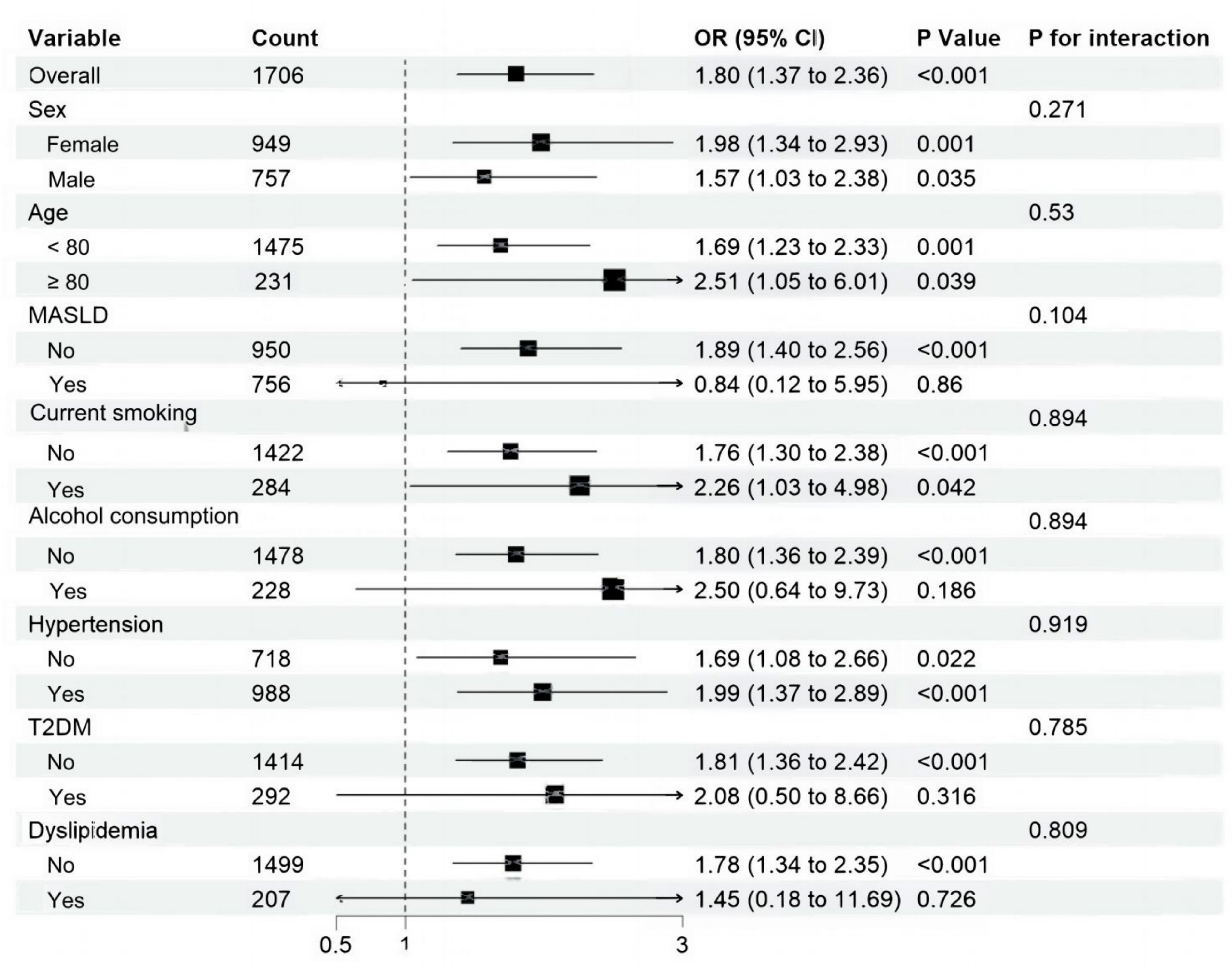
Subgroup analyses were performed to investigate the relationship between systemic inflammation response index and the prevalence of proxy-assessed sarcopenia across different subgroups. Model was adjusted for waist circumference, hemoglobin, alanine transaminase, aspartate aminotransferase, fasting plasma glucose, total cholesterol, total bilirubin, triglyceride, creatinine, erythrocyte count, high-density lipoprotein cholesterol, low-density lipoprotein cholesterol. MASLD, metabolic dysfunction-associated steatotic liver disease; T2DM, type 2 diabetes mellitus.

**FIGURE 3:**
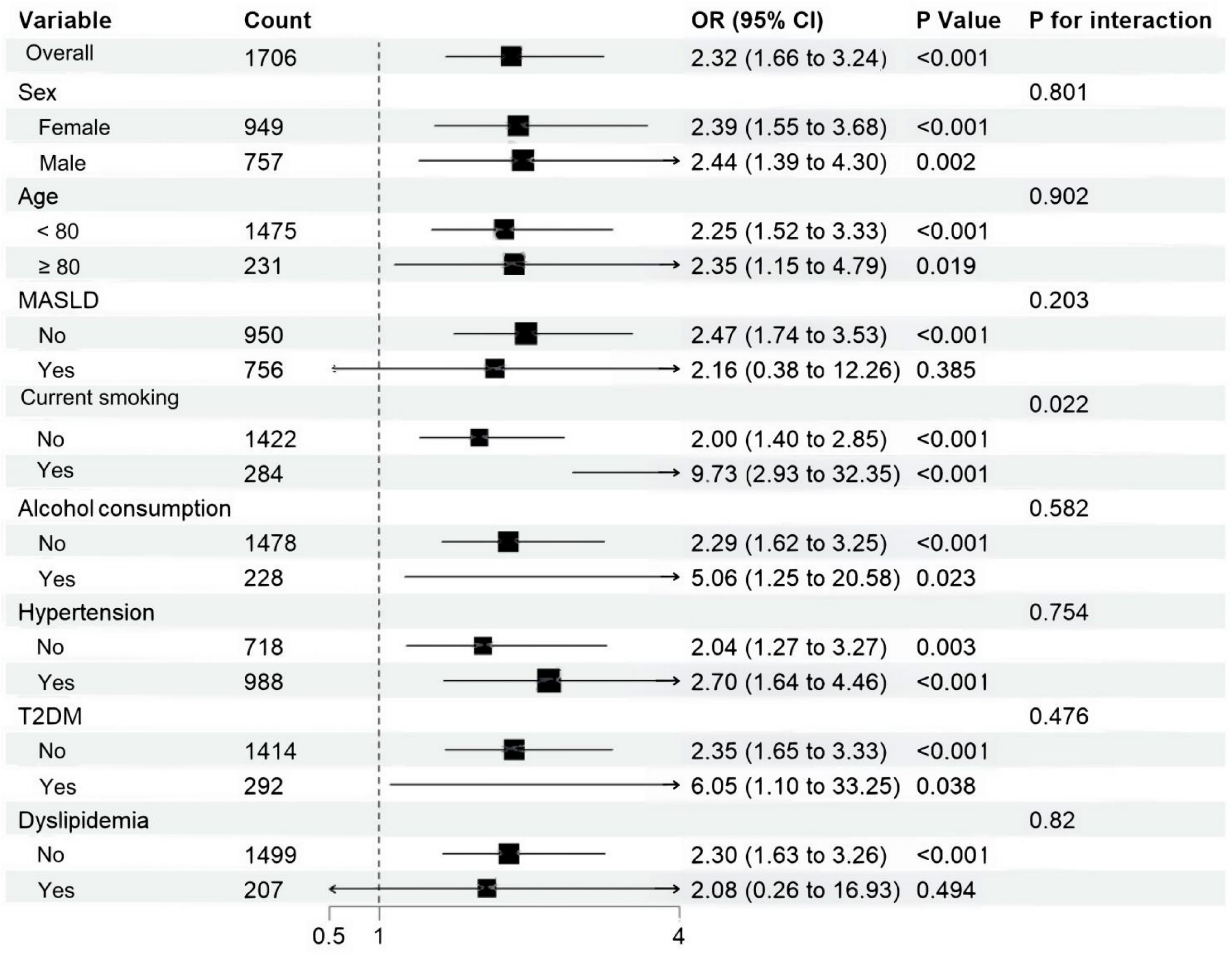
Subgroup analyses were performed to investigate the relationship between systemic immune-inflammation index and the prevalence of proxy-assessed sarcopenia across different subgroups. Model was adjusted for waist circumference, current smoking, alcohol consumption, hemoglobin, alanine transaminase, aspartate aminotransferase, fasting plasma glucose, total cholesterol, total bilirubin, triglyceride, creatinine, erythrocyte count, high-density lipoprotein cholesterol, low-density lipoprotein cholesterol. MASLD, metabolic dysfunction-associated steatotic liver disease; T2DM, type 2 diabetes mellitus.

Subgroup analyses were adopted to determine the associations of SIRI and SII with proxy-assessed sarcopenia across different subgroups. The populations were stratified by sex (female/male), age (≥80/<80), MASLD (no/yes), current smoking (no/yes), alcohol consumption (no/yes), type 2 diabetes mellitus (no/yes), hypertension (no/yes), dyslipidemia (no/yes). After adjusting for waist circumference, current smoking, alcohol consumption, hemoglobin, alanine transaminase, aspartate aminotransferase, fasting plasma glucose, total cholesterol, total bilirubin, triglyceride, creatinine, erythrocyte count, HDL-C, LDL-C, the positive association between SIRI and proxy-assessed sarcopenia remained statistically significant in most subgroups. Notably, no significant interaction was observed between SIRI and any of the stratification variables (P for interaction > 0.05 for all), indicating the robustness of this association across diverse subpopulations (Figure 2). Similarly, the association between SII and proxy-assessed sarcopenia was also significant in most subgroups after multivariable adjustment. However, a statistically significant interaction was detected between SII and current smoking status (P for interaction = 0.022). The association was markedly stronger among current smokers (adjusted OR = 9.73; 95% CI: 2.93–32.35) compared to non-smokers (adjusted OR = 2.00; 95% CI: 1.40–2.85), suggesting a synergistic or multiplicative effect of elevated SII and smoking on proxy-assessed sarcopenia risk (Figure 3).

## Discussion

Our study demonstrated that elevated SII and SIRI were significantly and independently associated with higher odds of proxy-assessed sarcopenia, providing novel evidence linking these two integrative inflammatory indices to proxy-assessed sarcopenia in a community-dwelling older population. After comprehensive adjustment for a wide array of metabolic and clinical confounders, both SII and SIRI demonstrated significant, independent, and dose-dependent associations with increased odds of proxy-assessed sarcopenia, as evidenced by quartile and trend analyses. Moreover, RCS modeling revealed that among the individual cell counts, only leukocyte count and neutrophil count showed a significant overall association with proxy-assessed sarcopenia, whereas the composite biomarkers–SIRI and SII–demonstrated statistically significant overall associations with the odds of proxy-assessed sarcopenia. Therefore, SII and SIRI, as integrative measures of inflammatory and immune status, may serve as more informative indicators than conventional differential leukocyte counts in reflecting the inflammatory state associated with proxy-assessed sarcopenia.

The overall prevalence of sarcopenia in the study based on community-dwelling older adults aged 65 years and over was 16.35%, and the results of the prevalence of sarcopenia were consistent with previous findings, which showed a prevalence of 9.9%–40.4% (6). Moreover, the prevalence of sarcopenia varied across different age groups. One previous study reported a prevalence of 38.5% among Chinese individuals aged ≥ 60 years (30); the higher prevalence compared to our finding (16.35%) may be attributed to differences in study population characteristics, diagnostic criteria, or regional factors. In addition, the prevalence of proxy-assessed sarcopenia increased with age in our study: it was 18.2% among those aged ≥ 70 years, 24.7% among those aged ≥ 75 years, and 34.6% among those aged ≥ 80 years. This trend indicates that age is a significant factor associated with sarcopenia. However, other chronic diseases may also influence its prevalence. Future studies should investigate the prevalence of sarcopenia across diverse disease populations and in broader community settings.

The high incidence of sarcopenia in the older adults has significant health implications. Sarcopenia is a progressive and complex disease associated with higher morbidity and mortality (31, 32), which significantly increase the financial burden and medical costs (4). Currently, there are no FDA-approved medications specifically designed to treat sarcopenia. Thus, exploring the pathogenesis of sarcopenia could help with drug development. A large body of evidence now suggests that low-grade chronic inflammation associated with aging may be a key factor in sarcopenia (9, 10, 33). Older adults experience a chronic low-grade inflammatory state during the aging process. Inflammatory cells and skeletal muscle cells can produce inflammatory cytokines, contributing to the development of sarcopenia. This condition manifests as decreased muscle mass, reduced muscle strength, and impaired physical function, which in turn increase the likelihood of adverse events (34). Our study showed positive associations between SIRI and SII with proxy-assessed sarcopenia, suggesting that an inflammatory state coexists with poor muscle strength and physical performance. Previous clinical studies indicated that reductions in limb skeletal muscle mass, strength, and physical performance were associated with high levels of pro-inflammatory cytokines such as IL-6, tumor necrosis factor-alpha, and C-reactive protein in men and women (35, 36). Our results demonstrated a positive association between higher SIRI or SII and the odds of proxy-assessed sarcopenia, which is consistent with previous studies reporting associations between pro-inflammatory cytokines and proxy-assessed sarcopenia (35, 36). SII and SIRI offer practical advantages over conventional cytokine measurements. As calculated from routine complete blood count parameters, they are readily available, inexpensive, and provide a dynamic snapshot of the systemic inflammatory and immune landscape.

While our study provides valuable insights into the association between SIRI and SII with proxy-assessed sarcopenia, several limitations should be acknowledged. First, the assessment of muscle mass relied on a population-specific prediction equation rather than direct imaging, and the use of a population-specific prediction equation may reduce the robustness and external comparability of the sarcopenia definition. Future studies employing gold-standard modalities such as magnetic resonance imaging (MRI), computed tomography (CT), or dual-energy X-ray absorptiometry (DXA) would allow for a more definitive evaluation of this relationship. Second, the lack of data on dietary intake and physical activity-related factors is a limitation of our study, and the omission of these variables may result in residual confounding and potentially bias in the observed associations; future studies should incorporate nutritional status and physical activity levels into the analytical models. Third, the cross-sectional design precludes the establishment of causal inferences. Longitudinal studies are needed to elucidate the temporal relationship between these inflammatory indices and the development of sarcopenia. Finally, further research is required to explore the underlying biological mechanisms and to evaluate the clinical relevance and generalizability of SII and SIRI across diverse populations and clinical settings.

## Conclusion

In conclusion, our findings demonstrate SIRI and SII as practical inflammatory biomarkers associated with proxy-assessed sarcopenia and reinforce systemic inflammation as a key modifiable target. This supports the potential value of these indices in identifying individuals at risk and guiding inflammation-focused prevention and management strategies in aging populations.

## Data Availability

The raw data supporting the conclusions of this article will be made available by the authors, without undue reservation.

## References

[1] Sanchez-Rodriguez D Marco E Cruz-Jentoft A. Defining sarcopenia: some caveats and challenges. *Curr Opin Clin Nutr Metab Care.* (2020) 23:127–32. 10.1097/mco.0000000000000621 31789867

[2] Cacciatore S Calvani R Marzetti E Picca A Russo A Tosato M et al. Physical performance is associated with long-term survival in adults 80 years and older: results from the ilsirente study. *J Am Geriatr Soc.* (2024) 72:2585–9. 10.1111/jgs.18941 38696049

[3] Beaudart C Demonceau C Reginster J Locquet M Cesari M Cruz Jentoft A et al. Sarcopenia and health-related quality of life: a systematic review and meta-analysis. *J Cachexia Sarcopenia Muscle.* (2023) 14:1228–43. 10.1002/jcsm.13243 37139947 PMC10235892

[4] Beaudart C Zaaria M Pasleau F Reginster J Bruyère O. Health outcomes of sarcopenia: a systematic review and meta-analysis. *PLoS One.* (2017) 12:e0169548. 10.1371/journal.pone.0169548 28095426 PMC5240970

[5] Fonseca G Dos Santos M de Souza F Takayama L Rodrigues Pereira R Negrão C et al. Discriminating sarcopenia in overweight/obese male patients with heart failure: the influence of body mass index. *ESC Heart Failure.* (2020) 7:84–91. 10.1002/ehf2.12545 31877587 PMC7083394

[6] Mayhew A Amog K Phillips S Parise G McNicholas P de Souza R et al. The prevalence of sarcopenia in community-dwelling older adults, an exploration of differences between studies and within definitions: a systematic review and meta-analyses. *Age Ageing.* (2019) 48:48–56. 10.1093/ageing/afy106 30052707

[7] Cho M Lee S Song SK. A review of sarcopenia pathophysiology, diagnosis, treatment and future direction. *J Korean Med Sci.* (2022) 37:e146. 10.3346/jkms.2022.37.e146 35535373 PMC9091430

[8] Tuttle C Thang L Maier A. Markers of inflammation and their association with muscle strength and mass: a systematic review and meta-analysis. *Ageing Res Rev.* (2020) 64:101185. 10.1016/j.arr.2020.101185 32992047

[9] Aluganti Narasimhulu C Singla D. Amelioration of diabetes-induced inflammation mediated pyroptosis, sarcopenia, and adverse muscle remodelling by bone morphogenetic protein-7. *J Cachexia Sarcopenia Muscle.* (2021) 12:403–20. 10.1002/jcsm.12662 33463042 PMC8061343

[10] Nardone O de Sire R Petito V Testa A Villani G Scaldaferri F et al. Inflammatory bowel diseases and sarcopenia: the role of inflammation and gut microbiota in the development of muscle failure. *Front Immunol.* (2021) 12:694217. 10.3389/fimmu.2021.694217 34326845 PMC8313891

[11] Qi Q Zhuang L Shen Y Geng Y Yu S Chen H et al. A novel systemic inflammation response index (Siri) for predicting the survival of patients with pancreatic cancer after chemotherapy. *Cancer.* (2016) 122:2158–67. 10.1002/cncr.30057 27152949

[12] Zhang S Cheng T. Prognostic and clinicopathological value of systemic inflammation response index (Siri) in patients with breast cancer: a meta-analysis. *Ann Med.* (2024) 56:2337729. 10.1080/07853890.2024.2337729 38569199 PMC10993763

[13] Huang P Mai Y Zhao J Yi Y Wen Y. Association of systemic immune-inflammation index and systemic inflammation response index with chronic kidney disease: observational study of 40,937 adults. *Inflammation Res.* (2024) 73:655–67. 10.1007/s00011-024-01861-0 38489048

[14] Ma M Wu K Sun T Huang X Zhang B Chen Z et al. Impacts of systemic inflammation response index on the prognosis of patients with ischemic heart failure after percutaneous coronary intervention. *Front Immunol.* (2024) 15:1324890. 10.3389/fimmu.2024.1324890 38440729 PMC10910016

[15] Zhang S Tang Z. Prognostic and clinicopathological significance of systemic inflammation response index in patients with hepatocellular carcinoma: a systematic review and meta-analysis. *Front Immunol.* (2024) 15:1291840. 10.3389/fimmu.2024.1291840 38469315 PMC10925676

[16] Han K Shi D Yang L Wang Z Li Y Gao F et al. Prognostic value of systemic inflammatory response index in patients with acute coronary syndrome undergoing percutaneous coronary intervention. *Ann Med.* (2022) 54:1667–77. 10.1080/07853890.2022.2083671 35695557 PMC9225721

[17] Hu B Yang X Xu Y Sun Y Sun C Guo W et al. Systemic immune-inflammation index predicts prognosis of patients after curative resection for hepatocellular carcinoma. *Clin Cancer Res.* (2014) 20:6212–22. 10.1158/1078-0432.CCR-14-0442 25271081

[18] Nie Y Zhou H Wang J Kan H. Association between systemic immune-inflammation index and diabetes: a population-based study from the Nhanes. *Front Endocrinol.* (2023) 14:1245199. 10.3389/fendo.2023.1245199 38027115 PMC10644783

[19] Guo J Xu R Liu R Lai W Hu C He H et al. Association between the systemic immune inflammation index and periodontitis: a cross-sectional study. *J Transl Med.* (2024) 22:96. 10.1186/s12967-024-04888-3 38263194 PMC10804475

[20] Ramezankhani A Tohidi M Hadaegh F. Association between the systemic immune-inflammation index and metabolic syndrome and its components: results from the multi-ethnic study of atherosclerosis (Mesa). *Cardiovasc Diabetol.* (2025) 24:78. 10.1186/s12933-025-02629-4 39955525 PMC11830208

[21] Tuzimek A Dziedzic E Beck J Kochman W. Correlations between acute coronary syndrome and novel inflammatory markers (systemic immune-inflammation index, systemic inflammation response index, and aggregate index of systemic inflammation) in patients with and without diabetes or prediabetes. *J Inflammation Res.* (2024) 17:2623–32. 10.2147/JIR.S454117 38707954 PMC11067916

[22] Dziedzic E Ga̧sior J Tuzimek A Paleczny J Junka A Da̧browski M et al. Investigation of the associations of novel inflammatory biomarkers—systemic inflammatory index (sii) and systemic inflammatory response index (siri)—with the severity of coronary artery disease and acute coronary syndrome occurrence. *Int J Mol Sci.* (2022) 23:9553. 10.3390/ijms23179553 36076952 PMC9455822

[23] Ma L Xiao H Zhang J Liu Y Hu L Chen N et al. Association between systemic immune inflammatory/inflammatory response index and hypertension: a cohort study of functional community. *Nutr Metabol Cardiovasc Dis.* (2024) 34:334–42. 10.1016/j.numecd.2023.09.025 38000992

[24] Hu S Song J Jiang H Wei B Wang H. Association between the dietary index for gut microbiota and cardiometabolic multimorbidity: systemic immune-inflammation index and systemic inflammatory response index. *Front Nutr.* (2025) 12:1591799. 10.3389/fnut.2025.1591799 40538590 PMC12176575

[25] Coşansu N Kara RÖ Yaldiz M Dikicier BS. New markers to predict the response to omalizumab in chronic spontaneous urticaria. *Dermatol Ther.* (2022) 35:e15589. 10.1111/dth.15589 35582853

[26] Chen L Woo J Assantachai P Auyeung T Chou M Iijima K et al. Asian working group for sarcopenia: 2019 consensus update on sarcopenia diagnosis and treatment. *J Am Med Directors Assoc.* (2020) 21:300–7.e2. 10.1016/j.jamda.2019.12.012 32033882

[27] Wen X Wang M Jiang C Zhang Y. Anthropometric equation for estimation of appendicular skeletal muscle mass in chinese adults. *Asia Pac J Clin Nutr.* (2011) 20:551–6. 22094840

[28] Jin Z Zheng L Sun C Xu B Guo X Zhang Y et al. More comprehensive relationship between egdr and sarcopenia in China: a nationwide cohort study with national representation. *Diabetol Metab Syndrome.* (2025) 17:97. 10.1186/s13098-025-01657-0 40122882 PMC11931793

[29] Unger T Borghi C Charchar F Khan N Poulter N Prabhakaran D et al. 2020 international society of hypertension global hypertension practice guidelines. *Hypertension.* (2020) 75:1334–57. 10.1161/hypertensionaha.120.15026 32370572

[30] Wu X Li X Xu M Zhang Z He L Li Y. Sarcopenia prevalence and associated factors among older chinese population: findings from the China health and retirement longitudinal study. *PLoS One.* (2021) 16:e0247617. 10.1371/journal.pone.0247617 33661964 PMC7932529

[31] Cruz-Jentoft A Sayer A. Sarcopenia. *Lancet.* (2019) 393:2636–46. 10.1016/s0140-6736(19)31138-9 31171417

[32] Petermann-Rocha F Ho F Welsh P Mackay D Brown R Gill J et al. Physical capability markers used to define Sarcopenia and their association with cardiovascular and respiratory outcomes and all-cause mortality: a prospective study from UK Biobank. *Maturitas.* (2020) 138:69–75. 10.1016/j.maturitas.2020.04.017 32471663

[33] Livshits G Kalinkovich A. Inflammaging as a common ground for the development and maintenance of sarcopenia, obesity, cardiomyopathy and dysbiosis. *Ageing Res Rev.* (2019) 56:100980. 10.1016/j.arr.2019.100980 31726228

[34] Pan L Xie W Fu X Lu W Jin H Lai J et al. Inflammation and sarcopenia: a focus on circulating inflammatory cytokines. *Exp Gerontol.* (2021) 154:111544. 10.1016/j.exger.2021.111544 34478826

[35] Haren M Malmstrom T Miller D Patrick P Perry H Herning M et al. Higher C-reactive protein and soluble tumor necrosis factor receptor levels are associated with poor physical function and disability: a cross-sectional analysis of a cohort of late middle-aged African Americans. *J Gerontol Ser A Biol Sci Med Sci.* (2010) 65:274–81. 10.1093/gerona/glp148 19812256 PMC2822280

[36] Alemán H Esparza J Ramirez F Astiazaran H Payette H. Longitudinal evidence on the association between interleukin-6 and c-reactive protein with the loss of total appendicular skeletal muscle in free-living older men and women. *Age Ageing.* (2011) 40:469–75. 10.1093/ageing/afr040 21565862

